# Experiences of an internet-delivered treatment of obesity: A qualitative study

**DOI:** 10.1016/j.invent.2025.100876

**Published:** 2025-09-26

**Authors:** Annika Imhagen, Stefan Jansson, Fredrik Söderqvist, Jan Karlsson, Marije Galavazi, Agneta Anderzén Carlsson

**Affiliations:** a University Health Care Research Center, Faculty of Medicine and Health, Örebro University, Örebro, Sweden; b School of Medicine, Faculty of Medicine and Health, Örebro University, Örebro, Sweden

## Abstract

• Internet-delivered obesity treatment can help change lifestyle habits
• Expectations for weight loss were not met
• Life circumstances made it difficult to commit to treatment
• Internet-delivered treatment is not for everyone

## 1 Introduction

The increasing prevalence of obesity is a growing public health concern. According to the World Health Organization (WHO), 16 % of the adult global population was living with obesity in 2022 (World Health Organization, 2025). Obesity is a chronic disease associated with increased risk of premature mortality due to several health complications, including type 2-diabetes, cardiovascular disease, and certain forms of cancer (Afshin et al., 2017). In addition, obesity often impairs quality of life (Kolotkin and Andersen, 2017).

Treatment options for obesity include lifestyle interventions, pharmacotherapy, and bariatric surgery (Yumuk et al., 2015). Lifestyle changes—such as adopting a healthier diet and increasing physical activity—are recognized as challenging but feasible for achieving long-term weight management (Varkevisser et al., 2019). Treatment programs based on cognitive behavioral therapy (CBT) can improve long-term outcomes, especially when combined with lifestyle interventions focusing on diet and physical activity (Kurnik Mesarič et al., 2023). CBT combines cognitive, behavioral and emotion-focused techniques with the goal of changing behavioral patterns (Hofmann et al., 2012). Behavioral strategies such as problem-solving and stimulus control can enhance adherence to lifestyle interventions in adults with obesity (Burgess et al., 2017).

It is challenging for healthcare to provide care to all people with obesity who could benefit from lifestyle interventions. Effective support takes time, and research has shown that at least 12 contacts between health care professionals and patients are required for better weight loss outcomes (Madigan et al., 2022). However, the limited time and resources in health care are often cited as barriers to individualized obesity treatment (Blackburn et al., 2015). Telephone or web-based interventions have been shown to improve health conditions and can be cost-effective compared with traditional care (Law et al., 2022). Results vary depending on the content, intensity, and length of the treatment (Beleigoli et al., 2019; Sorgente et al., 2017), but interventions that combine a digital program with face-to-face counseling have been shown to be more effective for weight loss than those without in-person counseling (Kupila et al., 2023). Moreover, it is difficult to determine which parts of a program actually help participants to change their behavior because different web-based programs use different methods. In a systematic review of web-based interventions by Shi et al. (Shi et al., 2022), programs lasted from 2 to 24 months and used a variety of approaches including online classes alone or combined with face-to-face treatment, e-mail counseling, computer-tailored feedback, or online chat. Commonly used components included behavioral self-monitoring, goal setting and behavioral feedback.

Understanding participants' perceptions and experiences is an essential component of program evaluation (Rand et al., 2019). In a review of consumer perspectives on mobile phone apps and text messaging for weight loss, participants described self-monitoring, goal setting, and feedback as benefits, while perceived barriers were related to technical and psychological issues, such as feelings of failure and guilt (Lyzwinski et al., 2018). A weight-management program using group video meetings was found to save time and money and provided peer support from other participants (Cliffe et al., 2021). One-way text message support as part of a community obesity program was found to be flexible and helped participants with focus and accountability; however, most of the participants wanted support for longer than the 4 months offered, and some wanted two-way communication (Lewis et al., 2021). Research specifically evaluating participants' experiences with Internet-delivered treatment (IDT) for obesity including interaction with a therapist is limited. Therefore, the aim of the present study was to describe participants' experiences of an IDT program for obesity (IDT—O) from the perspective of both completers and dropouts.

## 2 Methods

This study uses a qualitative descriptive design with an inductive approach and semi-structured individual interviews.

### 2.1 The Internet-delivered treatment program for obesity

The IDT-O is an individual treatment inspired by CBT and motivational interviewing created by health care professionals (referred to herein as “therapists”) at the obesity unit at a university hospital in Sweden. The program is based on a face-to-face group treatment for obesity that has been used clinically since 2010. The main goals of the IDT-O are to provide evidence-based knowledge about obesity, to support behavioral and lifestyle changes that can contribute to reducing body weight in the longer term and improving health status, and to mitigate the negative effects of weight stigma. The focus is not primarily on weight loss but on healthy lifestyle habits. Examples of established behavioral-change techniques used in the IDT-O are shaping knowledge, goals and planning, feedback and monitoring and self-belief. Patients access the online platform through a secure login from the Swedish National Health e-services at 1177.se. The 6-month program consists of 12 modules comprising 19,700 words (Supplementary Table 1), with a subsequent module being activated every 2 weeks. Each module consists of 4–5 sections with text (that can also be listened to), pictures and short videos. The modules end with tasks (e.g., setting a goal or registering steps/day for 7 days) to be completed before the next module is activated. Participants receive feedback on tasks from their assigned therapist via email, sent through the platform. If the patient has not been active for a week, an automatic reminder is sent to log into the program. Reminders are then sent weekly in case of inactivity. The therapists delivering the treatment in this study were two psychologists, a physiotherapist, and a dietitian.

### 2.2 Research trial

This study was conducted in the context of a 1-year randomized controlled trial (RCT) titled “Internet treatment for patients with obesity” (ClinicalTrials.gov ID NCT05149950), with the aim of evaluating the IDT—O. The RCT included 130 people (16 % men) aged 25–69 years with a body mass index (BMI) of 30–44.9 kg/m^2^ or 28–29.9 kg/m^2^ and at least one weight-related comorbidity. Preliminary data shows a dropout rate of 35 %. In the RCT, all participants received the IDT—O, including written communication with a therapist. Half of the participants (65 people) were randomized to the intervention group and received four video or face-to-face meetings with a therapist (before treatment and at 6, 12, and 18 weeks) in addition to the IDT—O. The control group consisted of the 65 participants who received IDT-O alone.

### 2.3 Participants

The participants in this interview study were recruited from the RCT described above. To obtain experiences from people with different characteristics (e.g., age, gender, education level), a purposive sampling was used when approaching the intervention-group participants who had completed the IDT—O. In addition, to understand the reasons for not completing the program, participants in both the intervention and control groups who did not complete the IDT-O (referred to herein as “dropouts”) were asked to participate in the study, using consecutive sampling. Twenty completers in the intervention group and a total of 10 dropouts (*n* = 4 from the intervention group and *n* = 6 from the control group) were included (Table 1). The dropouts had completed 2–5 modules before dropping out of the program. All participants had previous experience of trying to lose weight, and the desire to lose weight was their main reason for participating in the IDT—O. The participants wanted to lose weight because of concerns about their current or future health, to be able to be more physically active, or because they were dissatisfied with their appearance.

**Table 1:** Characteristics of interviewed participants and total population in RCT at baseline.

	Completers *n* = 20	Dropouts *n* = 10		RCT sample *n* = 130
		Intervention *n* = 4	Control *n* = 6	
Gender				
Male	5 (25 %)	1 (25 %)	1 (17 %)	21 (16 %)
Female	15 (75 %)	3 (75 %)	5 (83 %)	109 (84 %)
Age (years)				
Range	34–68	40–61	37–61	26–69
Median (IQR)	51 (15)	48 (17)	55.5 (17)	49 (14)
Highest education				
Secondary school	9 (45 %)	2 (50 %)	2 (33 %)	41 (32 %)
University	11 (55 %)	2 (50 %)	4 (67 %)	74 (57 %)
Missing				15 (11 %)
Occupational status				
Employed	18 (90 %)	3 (75 %)	5 (83 %)	101 (78 %)
Otherᵃ	2 (10 %)	1 (25 %)	1 (17 %)	29 (22 %)
Missing				13 (10 %)^c^
BMI				
Range	29.7–41.3	31.7–37.3	31.3–42.8	28.7–45.1
Median (IQR)	35.2 (4.8)	34.6 (4.8)	32.4 (8.2)	35.4 (6.8)
Obesity				
Class I (BMI 30.0–34.9)	9 (45 %)ᵇ	2 (50 %)	4 (66.7 %)	59 (45 %)ᵇ
Class II (BMI 35.0–39.9)	10 (50 %)	2 (50 %)	1 (16.7 %)	39 (30 %)
Class III (BMI ≥40)	1 (5 %)		1 (16.7 %)	32 (25 %)

a Student, retired, or on sick leave.

b Including 2 and 7 persons respectively with a BMI under 30.

c Multiple answers were possible, thus resulting in >100 %.

### 2.4 Interview procedure

**Fig. 1:**
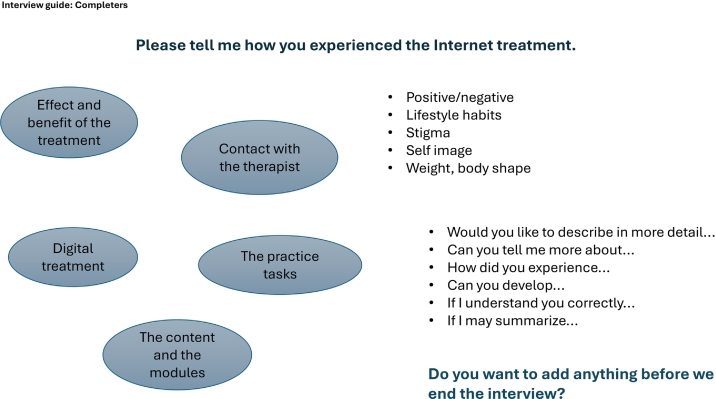
Interview mind map for program completers.

**Fig. 2:**
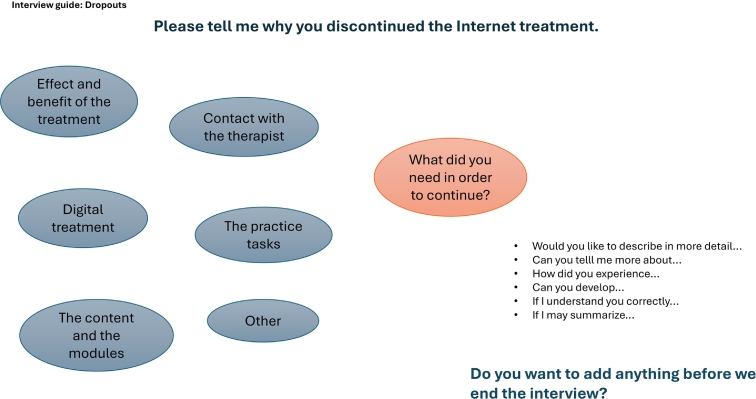
Interview mind map for program dropouts.

Qualitative data were collected through individual semi-structured interviews between April 2022 and September 2023. Two topic mind maps with open-ended probing questions, one for completers and one for dropouts (Fig. 1, Fig. 2), were developed based on clinical experience and the literature. Participants were invited to participate in an online interview either after completing the program or when dropping out. Both groups were asked to share their experiences with the IDT-O and dropouts were also asked to elaborate on their reasons for dropping out of the program. The interviews were conducted by the first author (AI), who is a PhD student and a registered nurse with experience working with patients with obesity. No one in the research team had a previous or ongoing relationship with the participants. The interviews were conducted via a secure online meeting platform and lasted between 16 and 45 min (mean 27 min). They were recorded with the participants' consent and later transcribed verbatim by a professional transcription agency.

### 2.5 Data analysis

The qualitative content analysis was inspired by the steps described by Graneheim and Lundman (Graneheim and Lundman, 2004). The recorded interviews were first listened to; the interviewer validated the transcriptions and corrected any errors reading several times to get an overall impression of the material. The transcriptions were then imported into NVivo software (QSR International Pty. Ltd., 2020) to manage the data. Meaning units (sentences and paragraphs) related to the study aim were identified by the first author and labeled with a code that was close to the text and represented the content (e.g., “the program was thought provoking,” or “it's easy to fall back into old habits”). All authors read three of the transcribed interviews to validate the coding of these three interviews. The codes were inductively compared for similarities and differences and finally eight mutually exclusive categories were identified. This involved a careful iterative process of grouping and regrouping the content to create mutually exclusive categories that answered the research question and were close to the text (Graneheim and Lundman, 2004). The underlying meaning of the categories was finally formulated in one main theme and two subthemes. The categories captured the participants' statements at a manifest level, while the themes were an interpretation of the participants' stories at a higher level of abstraction (Graneheim et al., 2017).

Trustworthiness was established according to the strategies described by Shenton (Shenton, 2004) regarding credibility, transferability, dependability and confirmability. There were repeated discussions between two of the authors (AI and AAC) about selecting appropriate meaning units and assessing similarities and differences within and between categories, and the analysis was reviewed by all the authors (Graneheim and Lundman, 2004). The different professions and backgrounds of the authors (two nurses, two physicians, one psychologist, one public health scientist) enriched the analysis and results (Shenton, 2004). Four of the authors had experience working with patients with obesity. Appropriate quotations can enhance the credibility of a finding (Graneheim and Lundman, 2004); therefore translated quotations from the transcribed interview text were used to support and illustrate the interpretations. The Consolidated Criteria for Reporting Qualitative Research (COREQ) checklist was used to ensure explicit and comprehensive reporting (Tong et al., 2007).

### 2.6 Ethics

The study was conducted in accordance with the Helsinki Declaration (World Medical Association, 2024) and was approved by the Swedish Ethical Review Authority (registration nos. 2021–03726, 2023–02869-02). Informed written consent was obtained from all participants, who were informed that they could stop the interview at any time.

## 3 Results

The analysis yielded one main theme: “The program has the potential to make a difference” and two subthemes: “This was a first step toward sustainable changes in my life” and “It did not turn out exactly as I had imagined” (Table 2).

**Table 2:** Overview of the main theme, subthemes, and categories.

Main theme	Subthemes	Categories
The program has the potential to make a difference	This was a first step toward sustainable changes in my life	My overall impression was generally positive I started thinking in a new way I changed lifestyle habits I will continue on this path
	It did not turn out exactly as I had imagined	Not everything was what I expected I felt alone and struggled on my own Circumstances in life made it difficult for me to commit Technology was an obstacle for me

### 3.1 The program has the potential to make a difference

The main theme suggests that the IDT-O had potential to help participants begin to change their lifestyle habits by thinking and acting in new ways. However, several participants stated that the program was not what they had expected and that there were obstacles to overcome. Some realized that this program was not for them and moved on to find other help.

### 3.2 This was a first step toward sustainable changes in my life

The therapists were perceived as supportive by participants, and the information in the program was considered relevant. For some, participation in the program led to a change in mindset and the adoption of new habits.

#### 3.2.1 My overall impression was generally positive

The therapists were described as understanding and caring, providing timely and relevant feedback. In the intervention group, questions that could not be answered in writing were followed up by video meetings. The therapists offered advice on various issues, provided explanations, and encouraged participants to engage in physical activity, for example. Contact with a therapist was considered essential, as it was important for participants to feel recognized and to have someone to discuss ideas with. One participant said:

The thing was that you had someone to bounce ideas off… If you do it by yourself, you don't have anyone to refer to but yourself. Here, you can run ideas by someone and get some advice. You feel that someone somehow sees you. (Completer #35).

The IDT-O was perceived as inspiring and as providing relevant information on topics such as diet and physical activity. The participants said the digital format saved time and let them participate at their own pace and time. They liked that they could read or listen to the text, and appreciated learning to take one step at a time and the program's focus on both cognition and behavior:

What I liked about it was that it was… you took one step at a time, and it wasn't like, “You need to stop eating, now you can only eat this.” It was really focused on the mental aspect as well, “Think like this,” before you start making changes to your diet. It was like a gentle start, and I thought that was good. (Dropout #15).

#### 3.2.2 I started thinking in a new way

The participants said the program was thought provoking and helped them contemplate and reflect on their own situation. Through self-reflection, they became more aware of their habits, diet, and eating behaviors. They began to accept themselves and recognize obesity as a chronic disease, becoming more forgiving of themselves in the process of changing their mindset:

I'm possibly a bit more forgiving of myself…and not feeling any guilt, shame, regret, or something when I have that extra evening sandwich because I'm actually hungry. (Completer #40).

The participants also began to prioritize their own health and well-being. They realized that feeling good and having healthy habits were more important than focusing solely on the number of kilos on the scale.

Despite adopting a new way of thinking, the participants talked about the challenge of applying knowledge about healthy lifestyle habits to themselves. One participant expressed sadness about letting go of old habits, such as using food for comfort. Others said that it was easy for them to revert to old habits and fall back into the same rut as before. They knew exactly what to do but sometimes lacked the time or energy to do it or prioritized their family's needs over their own. They wanted help understanding themselves and their behavior:

Even though I have the knowledge of what I need to do…I can't bring myself to do it anyway. (Dropout #26).

#### 3.2.3 I changed lifestyle habits

Even though it required time and effort, the participants reported adopting new lifestyle habits during the program. They noted that one of the major benefits of the IDT-O was that it increased their physical activity. This led to higher energy levels, improved mobility, and better ability to perform daily tasks:

I'm obviously more energetic, but I don't really see that I've lost weight. But having energy is the real difference, it's nice to be able to do more. The goal is kind of having the strength…before, I came home and just fell asleep on the couch, but now I can go out and be active with the kids. (Completer #27).

The participants also reported changing their dietary habits, reducing their intake of energy-dense foods, sweets, and snacks, and increasing their vegetable consumption. The IDT-O prompted them to change their eating behavior as well; they began eating more regularly, including breakfast, and limiting the amount by eating smaller portions and avoiding extra servings. They also ate more slowly and developed strategies for managing cravings. The participants said that this new approach to eating—such as not overeating—led to increased well-being.

#### 3.2.4 I will continue on this path

The end of the IDT-O program after 6 months was not viewed by the participants as the end but rather as the continuation of a healthier life. The participants said that they planned to maintain their changed lifestyle habits after the program and were motivated to continue losing weight. They understood it would be challenging but were determined to persevere for the sake of their health. Participation in the IDT-O gave them the sense of having made progress and the hope of being able to continue:

Well, for my part, it feels like I've…come a bit along the way, anyway. So I feel like I can, definitely, I hope, continue and then also be able to adapt to reality later. (Completer #6).

The participants recognized that change takes time and acknowledged that there would be setbacks they would have to deal with. The program provided useful tools; one dropout even saved material from the modules for future use:

I've saved…the sheet with the plan and goals, and I have a checklist for healthy food choices on a day and pitfalls. So, I've saved it, and I can use it when I've found enough of my own motivation. (Dropout #8).

### 3.3 It did not turn out exactly as I had imagined

While the IDT-O helped some participants make lifestyle changes, they expressed that it did not meet all their needs. Some were disappointed that there was so little focus on weight loss, and some experienced challenges related to the program.

#### 3.3.1 Not everything was what I expected

The participants expected weight loss, and several dropped out because they felt the program did not offer enough support to achieve this goal. They wanted to weigh themselves regularly—as both a carrot and a stick—and to report their weight and lifestyle changes to their therapist in person. They said it was important to be accountable to someone:

But I would have needed more active visits, physical visits, and…a little more…I will not say pressure, but to have something to show, to have someone to meet, and explain and discuss with. Yes, but positive pressure, not negative. I want to perform if I'm going to meet this person and talk and kind of discuss what I have done and maybe show what I've written and so on. (Dropout #15).

The participants identified specific expectations that were not met, such as help with emotional eating or access to recipes for healthy meals. Their need for continued support and follow-up after the IDT-O was also expressed:

So, where do you go from here, where can you find more help? I hope it will be…if you end up in a primary healthcare center or with a therapist or a dietitian…that's where I want to be and get help, more help. Because, obviously, I can't do this by myself. (Completer #28).

Many participants wanted further weight loss support after the program, which some sought independently, such as obtaining a referral for bariatric surgery. Others suggested combining the program with other forms of support, such as medication or concurrent participation in Weight Watchers.

#### 3.3.2 I felt alone and struggled on my own

The program left some participants feeling isolated, struggling to maintain focus, and left to fend for themselves:

You were pretty much left to yourself. And it is quite tough when you are struggling…to do everything yourself. You would have needed another kind of support then. (Completer #32).

The dropouts in the control group wanted more personal contact with their therapist, and some completers wanted additional meetings with their therapist. Several participants would have preferred to see their therapist in person rather than online. Some said that, during the program, they realized they needed support from others in similar situations to make changes:

I probably want to have some people around me, to hear a little bit about what others have done, or thought, or felt, so you can maybe identify yourself [with someone]…in some way… So that would've been, it would've given me much more, if I had got to meet others as well. (Dropout #72).

#### 3.3.3 Circumstances in life made it difficult for me to commit

Several participants left the IDT-O due to unexpected life events, such as illness in the family or job changes. The participants (both completers and dropouts) described the timing of the IDT-O as inconvenient for their current life situation, with some speculating that they would have continued if life had been different:

A separation and other things happened right around the same time. So it was just really bad timing in my life, simply put… And I felt like, well, this probably would've worked at another time in my life. (Dropout #26).

The participants described physical health issues—such as hip injuries, abdominal problems, or breathing difficulties—as obstacles to completing the program. Despite facing these challenges, some were able to complete the program to the best of their ability. Several felt that they lacked the time or commitment to complete the program. They found it difficult to integrate the IDT-O into their daily lives, experienced a loss of motivation (especially toward the end of the program) and rushed through the tasks. One participant commented:

It wasn't worth putting in the effort—because what I did was sloppy. I was given tasks like “Just read through this.” Yes, so I read through it and answered the questions. That's how it was, so it was me who didn't feel engaged in the whole thing. (Dropout #54).

The participants found it challenging to catch up on modules when they were ill, during holidays, and at other times. They also found it difficult to find time for self-reflection in their daily lives.

#### 3.3.4 Technology was an obstacle for me

The Internet platform used in the program was described as time-consuming to use, cumbersome and not user friendly. The participants were sometimes reluctant to log on to the platform because of the hassle involved:

The app itself was a bit cluttered, and it was hard to find what you were looking for. That's probably the thing I had the most negative things to say about. So it also makes you a bit resistant to opening it because you have to navigate to find your way. (Completer #39).

The limited mobile phone compatibility was described as a negative factor, and the participants using a mobile phone stated that the program would probably have worked better on a computer. The excessive number of automatic reminders from the Internet platform irritated some participants. Others pointed out that a reliable Internet connection was necessary to be able to complete the program.

## 4 Discussion

The purpose of this study was to describe the experiences of participants in an IDT program for people with obesity. Both completers and dropouts were interviewed, providing a broad and nuanced picture of the program. The results reflect different views and are characterized by the fact that both completers and dropouts had both positive and negative experiences with the program. This is illustrated in the two subthemes stating that the IDT-O can be a first step toward sustainable lifestyle changes, but that it did not turn out as expected. Some persons had objections to the content of the program and some to the format.

The results show that contact with the therapist was essential. The therapist provided support, advice, and feedback, and made the participants feel recognized. This finding is consistent with previous studies on IDT for obesity (Renouf et al., 2015; Vincze et al., 2018) and for other conditions (Asplund et al., 2019; Westas et al., 2022). Although most of the participants in our study were satisfied with the contact with their therapist, some wanted more frequent contact, ideally face to face. This result aligns with previous qualitative research in which participants requested increased face-to-face contact and communication (Lunde et al., 2022; Sayar et al., 2023).

Some participants said they would have benefited from having someone to check up on them. This result is partly consistent with a study by Renouf et al. (Renouf et al., 2015) on nurse support in an IDT weight-management intervention, which reported two groups of participants: those who were autonomously motivated and those who were more externally motivated. The participants in our study were similar to the latter group, as they expressed that they wanted to be accountable to their therapist—a finding that also aligns with those of other studies (Asplund et al., 2019; Sayar et al., 2023). Nevertheless, while support may be helpful during the treatment, what happens after the treatment, when patients are on their own? More research is needed on what level of support is needed and how to best help patients to become independent in changing and maintaining healthy lifestyle habits (Kupila et al., 2023).

Some were disappointed by the lack of focus on weight loss in the IDT-O and dropped out because they felt they were not getting the help they expected. The goal of the program is not primarily to lose a lot of weight during the 6-month treatment but to support lifestyle changes that may affect weight in the longer term, possibly several years. The therapists may have been insufficiently clear when explaining the program; or, some participants may not have understood the goals of the program, which was a reason for dropout in a study by Johansson et al. (Johansson et al., 2015). There is often a discrepancy between what patients expect in terms of weight loss and what is actually feasible, and it is important to help patients set realistic and achievable goals (Pétré et al., 2018).

The digital format was perceived as flexible and convenient, allowing participants to complete the program in their own time and at their own pace. This was also the view of participants in other studies on IDT (Westas et al., 2022; Lunde et al., 2022). However, some of the participants in our study found that IDT did not suit them. They felt alone and lacked contact with other participants with whom to share experiences and thoughts. For these individuals, group treatment or connecting with other participants through an online forum may have worked better. Technology was a barrier for some participants, as has been shown in other studies (Nomeikaite et al., 2023). IDT is becoming increasingly common in several areas of healthcare, which is good for accessibility, but it is important to recognize that it is not appropriate for all patients.

During the recruitment period, everyone who expressed interest in the IDT-O and met the inclusion and exclusion criteria was invited to participate in the RCT. The dropout rate in the study was greater than expected; one explanation may be that we included people for whom the IDT-O was not suitable at that time in their lives. The capacity was about 30 participants in treatment at a time. This meant that some potential study participants had to wait up to 12 months before starting the program. During this waiting period, motivation, commitment or their life situation might have changed. In a study that interviewed therapists offering IDT for depression, the results showed that IDT is not the right treatment for every patient and that it is important to assess which patients would benefit from the program (Børtveit et al., 2023). Kupila et al. (Kupila et al., 2023) emphasized that IDT for obesity should be offered to those who are most likely to benefit from it, which requires knowing what works for whom. Patel et al. (Patel et al., 2020) also emphasized this in a review, recommending the inclusion of an initial assessment of patients' expectations, preconceptions, and preferences. Participants in the IDT-O need to be able to concentrate when using a computer or mobile phone. A capacity for reflection, a willingness to try new things and work on your own without peer-support and perseverance are other important qualities. The participant's life situation is also very important. For example, it is difficult to engage in treatment if you are feeling mentally unwell or are stressed for various reasons. Finally, the participant must be aware that the IDT-O requires time and behavioral and emotional commitment.

### 4.1 Strengths and limitations

One limitation of this study is that control-group completers were not interviewed. Apart from the video meetings with a therapist, the control group received the same treatment as the intervention group, from whom we received substantial feedback. It is possible that the people who agreed to be interviewed were more motivated, and there is a risk that some—perhaps more negative—opinions were not captured. Nevertheless, the number of interviews was considered sufficient to capture a rich variety of experiences of the IDT—O. As with most qualitative studies, the ability to generalize the findings to other groups and settings is limited, for example to other cultures or health care systems. However, the fact that the results of this study share similarities with other qualitative studies on IDT makes it likely that some parts may be transferable. The interviewer was not a professional from the obesity unit, which hopefully allowed the participants to speak freely. The interviews were conducted within 1–3 months of completion of or withdrawal from the IDT—O, which provided conditions for the participants to recall their experiences and perceptions of the program.

## 5 Conclusions

In conclusion, the results of this study show that the IDT-O has the potential to make a difference in the lives of people with obesity and that it can be a first step toward changing lifestyle habits. However, for some participants, the program did not meet their expectations and additional help was desired. The results indicate that it is important to provide accurate information and convey realistic expectations of the program, considering the patients' current life situation. There is also a need to improve the procedures for assessing which patients are suitable for a treatment like this.

The following is the supplementary data related to this article.

Supplementary Table 1 Main content of the 12 treatment modules in the Internet-delivered treatment program for obesity (IDT—O) ^1^. Supplementary Table 1

## Funding

Open access funding provided by 10.13039/501100003509Örebro University. The authors received financial support from University Health Care Research Center, Region Örebro County, Örebro, Sweden. The study was financed by grants from the Swedish state under the agreement between the Swedish government and the county councils, the ALF-agreement (OLL-999403).

## Declaration of competing interest

The authors declare the following financial interests/personal relationships which may be considered as potential competing interests: Marije Galavazi reports a relationship with Novo Nordisk Inc. that includes: consulting or advisory and speaking and lecture fees. Marije Galavazi reports a relationship with Eli Lilly that includes: speaking and lecture fees. If there are other authors, they declare that they have no known competing financial interests or personal relationships that could have appeared to influence the work reported in this paper.

## Glossary

BMI: Body mass index
CBT: Cognitive behavioral therapy
COREQ: Consolidated Criteria for Reporting Qualitative Research
IDT: Internet-delivered treatment
IDT-O: Internet-delivered treatment program for obesity
