# Supplementary material for: Experiences of an internet-delivered treatment of obesity: A qualitative study
Source: Internet Interv. 2025 Sep 26;42:100876. doi: 10.1016/j.invent.2025.100876 (PMC12554118; doi:10.1016/j.invent.2025.100876)
Supplement: Supplementary Table 1 — Main content of the 12 treatment modules in the Internet-delivered treatment program for obesity (IDT—O) 1. [file mmc1.docx]

**Supplementary Table 1**

Main content of the 12 treatment modules in the Internet-delivered treatment program for obesity (IDT-O) ^1^

| *Module 1. Introduction*  Main content: The structure of the program. What to expect. My goal. My logbook.  Task: Answer questions to “My goal, my plan.” |
| --- |
| *Module 2. Cornerstones of healthy lifestyle habits and the disease obesity*  Main content: The cornerstones of healthy lifestyle habits. Obesity. What causes obesity? Consequences of obesity.  Task: Food diary for 4 days. |
| *Module 3. Setting goals*  Main content: Functional goals. The SMART model². Weight versus health.  Task: Formulating goals in “My goal, my plan.” |
| *Module 4. How to know what to eat?*  Main content: Nutrition. Meal rhythm, Portion size.  Tasks: Register meal rhythm and intake of vegetables. |
| *Module 5. Change process*  Main content: To register is the first step. Stages of the change process. Start, start over, and start over again. Risk situations.  Task: Identify and handle risk situations. |
| *Module 6. Physical activity*  Main content: Reduce sedentary time. Health-promoting advice for everyone. Facts about physical activity. What happens in your body? Obstacles to physical activity.  Tasks: Register steps/day. Activity diary. |
| *Module 7. Balance in life*  Main content: Balance in life. What is stress? What happens in the body during stress? Sleep. Stop and reflect.  Task: Try the mindfulness exercise “Breathe” for a week. |
| *Module 8. Eating behavior—how do I eat?*  Main content: Unconscious eating. Emotional eating. Hunger—satiety. Handling cravings.  Tasks: Food diary (based on Hunger—satiety). Situational analysis (based on e.g., emotional eating). |
| *Module 9. Problem-solving*  Main content: What is problem-solving? Problem-solving in 6 steps.  Task: Implement problem-solving according to model. |
| *Module 10. Body ideals and norms in society*  Main content: Norms in society. Negative thoughts. Body activism. Self-compassion.  Tasks: Characteristics of someone I like. What do I like about my body? |
| *Module 11. Success factors*  Main content: Success factors in weight loss. Maintenance plan.  Task: Fill in the maintenance plan “The traffic light”³ |
| *Module 12. This is my life*  Main content: I decide. Summary.  Tasks: Repeat registering according to the cornerstones of healthy lifestyle habits: food diary, meal rhythm, intake of vegetables, and physical activity. Compare with previous registrations. |

^1^ Participants work with each module for 2 weeks, completing tasks after each module on which they receive written feedback from the therapist. Treatment lasts for 6 months. ² The SMART (Specific, Measurable, Achievable, Relevant, and Time-bound) model: A method for setting goals. ³ “The traffic light”: Strategies to maintain healthy lifestyle habits and a stable weight. Green light: This is how I know I am on the right track. Yellow light: This is how I know I am moving away from my goals. Red light: This is how I notice that I have lost focus.
